# Radial Water Loss Assay in Rice Roots Grown in Different Media

**DOI:** 10.21769/BioProtoc.5845

**Published:** 2026-10-05

**Authors:** Lucas L. Peralta Ogorek, Samuel Jamieson, Bipin K. Pandey

**Affiliations:** Plant and Crop Science Department, School of Biosciences, University of Nottingham, Nottingham, UK

**Keywords:** Abiotic stress, Gas diffusion, Lignin, Suberin, Outer apoplastic barrier, Water vapor

## Abstract

Plants growing in soil are exposed to several environmental stresses, such as soil compaction, flooding, or drought, and need to quickly acclimate to these conditions to survive. Such acclimations include suberization and lignification of the outer and inner parts of the root. Visualization of suberin and lignin depositions in roots can be done with several well-established staining techniques followed by microscopy, but these methods are mostly qualitative. Radial water loss assays provide a quick, robust, and quantitative method to evaluate the formation of suberized and/or lignified outer apoplastic barriers and how tightly they regulate water loss. Here, we provide a detailed step-by-step protocol, from harvesting the roots from soil conditions and cleaning them, to preparing them for water loss measurements, while using basic lab equipment. Since the data obtained is water loss as mass, quantitative and statistical comparisons can be performed. Finally, while in this protocol roots were grown in soil, the method can be applied to roots growing in hydroponics, agar plates, or any other growth conditions that allow non-destructive harvesting of roots. In summary, the protocol provides a fast and reliable method to assess differences in outer apoplastic barrier development of root tissues, allowing for fast root tissue screenings while obtaining quantitative data without expensive or highly specialized equipment.

Key features

• Ideal for root tissues of similar diameter.

• Few preparation steps, and only simple lab equipment is needed.

• Data can be obtained in three days.

## Graphical overview


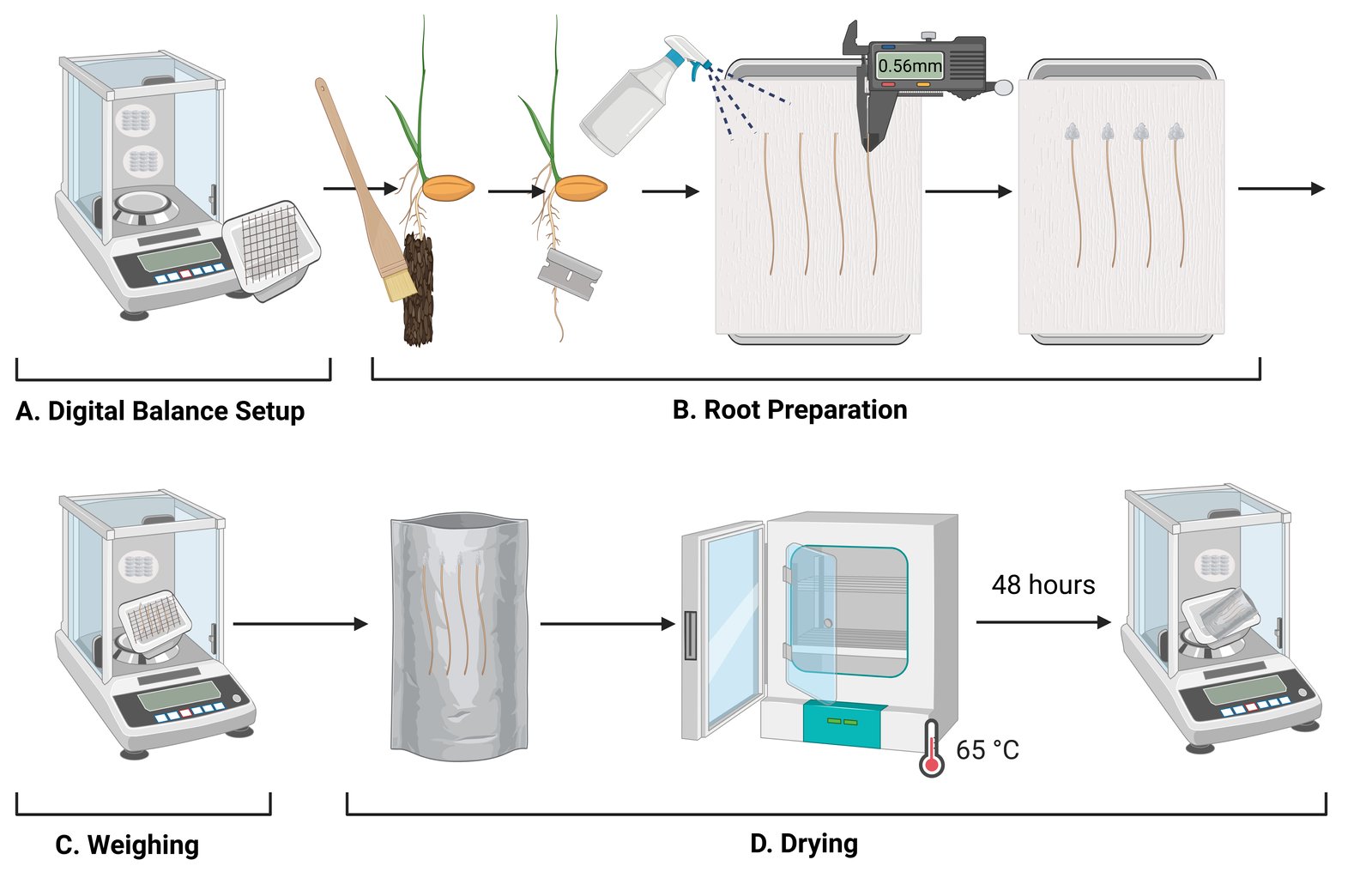


**Overview of the radial water loss assay in rice roots**

## Background

Climate change is the driving force behind the shift of rain patterns across the globe, subjecting areas to flooding or drought conditions that were not prone to such stresses before, or even exacerbating water stress conditions in those already experiencing them [1]. Moreover, the growth of the global population is increasing the pressure on agricultural areas in terms of food production, often requiring the use of heavy machinery [2,3]. This machinery, while necessary for food production, can cause compaction of the farmland soil, reducing root penetration and, ultimately, impacting the yield of such crops [4]. Roots are evidently exposed to a wide array of challenging soil and environmental conditions, which can be overcome with the suberization and lignification of the outer root tissues and the formation of outer apoplastic barriers [4,5].

Several well-established methods allow the assessment of suberized and lignified tissues with various staining fluorescent dyes, such as fluorol yellow (suberin) or basic fuchsin (lignin) [6]. While these methods provide useful spatial indication of suberized and lignified tissues of the roots, they remain mainly qualitative and do not allow the assessment of the strength or tightness of such root tissues to prevent gas diffusion in relevant contexts, such as oxygen loss during soil flooding (hypoxic stress [7]), water loss during soil compaction, or water deficiency stress [8].

These limitations are solved by radial water loss measurements from root tissues. Suberized and lignified outer root tissues become hydrophobic and limit the loss of water (as well as other gases) from the root to the external environment [5]. Moreover, the water loss measured as mass provides quantitative data with good temporal resolution (in the range of seconds). Other factors can drive the rate of water loss, such as root thickness; therefore, a comparison between roots should aim for similar diameters. While normalizing the data to root diameter is possible, it might not be adequate if the diameters differ greatly [7]. Alternatively, mutants deficient in suberization/lignification of tissues and/or formation of outer apoplastic barriers [8,9] could be applied to avoid this issue, provided that the differences in root diameters between the wild type and mutants remain constant across treatments.

Radial water loss data obtained can be used to compare the tightness of the outer apoplastic barriers. Suberized and/or lignified outer apoplastic barriers reduce radial water loss (e.g., non-compacted vs. compacted soil conditions in rice and *mhz5* [8], and well-drained vs. stagnant conditions in rice species from diverse natural habitats [1]). This method has been tested between species (e.g., rice, wheat) as well as with various diameters of agar cylinders to mimic roots [10]. Finally, the method was applied to roots without or with a root exodermis, including maize, wheat, Rhodes grass, or sorghum, and the results showed that the presence of an exodermis reduces radial water loss [11].

## Materials and reagents

**Biological materials**

1. Rice line cv Nipponbare

Rice seeds were dehusked and surface-sterilized using 25% sodium hypochlorite with 0.01% Triton for 5 min, followed by five rinses in sterile water. The seeds were then incubated in moist filter paper at 28–30 °C in the dark for 48 h to allow for germination. Germinated rice seedlings were transferred into three-dimensionally printed soil columns (15 cm height, 3.3 cm diameter) for 3 days. The soil used was loamy sand soil, and the bulk density was 1.2 g/cm^3^. The soil was sprayed with water (roughly adding 7% of moisture to the dry soil) before preparing the soil columns. Then, the columns were hydrated via capillarity from the bottom of the column for at least 2 h and then gravity-drained to reach field capacity. For more details, see [3]. A small hole for each seed was made in the topsoil before transferring the seedlings to provide space for the already emerged radicle. Once transferred to the soil columns, rice seedlings grew under the following conditions: 12/12 h photoperiod, 300 μmol m^2^/s light, and 70% relative humidity. To calculate the bulk density, we used the following formula:

Bulk density (g/cm^3^) = dry soil (g) / volume of soil container (cm^3^).

In this case, as the soil container is a cylinder, the volume is:

Cylinder volume (cm^3^) = π × radius^2^ (cm^2^) × height (cm)

**Laboratory supplies**

1. High-vacuum grease (Dow Corning, catalog number: 1597418); store at room temperature, in a cool, dry place, and ensure that the grease lid is tightly closed once finished using

2. Silica gel self-indicating (Fisherbrand^TM^, catalog number: S/0761/60); store at room temperature, in a cool, dry place, and tightly close the lid of the container after using

*Note: Any silica gel is acceptable as long as it can maintain the relative humidity constant inside the balance chamber throughout the measurements.*

3. Aluminum foil (Scientific Laboratory Supplies, catalog number: ALU1011)

4. Weighing boat (Sigma-Aldrich, catalog number: HS1420AA)

5. Soft hairbrush 4 mm (Scientific Laboratory Supplies LTD, UK); as long as the brush is soft, other sizes can be used

6. Razor blades (Wilkinson Sword, UK)

7. Tweezers (Fisherbrand^TM^ Dumont #5 Fine Tip Tweezers, catalog number: 15483542)

8. Thin nylon mesh (nylon dress net fabric, Fabric Land, UK); any mesh of a material that does not absorb water (e.g., nylon, metal) can be used

9. Tea bags (round or square, e.g., Bird and Blend, UK, catalog number not available)

10. Tape (e.g., 3M Micropore Surgical Tape, Scientific Laboratory Supplies, catalog number: 4702)

11. Spatula (VWR International LTD, catalog number: RSGA830.500) (there is no specific spatula for this)

12. Ruler (Scientific Laboratory Supplies, catalog number: GW510) (there is no specific ruler for this)

13. Paper towels (e.g., Kimberly-Clark^TM^ Scott^TM^ Slimfold Hand Towels, Fischer Scientific, catalog number: 11715254); any paper towel is acceptable provided it is soft and kept moist while covering root segments

## Equipment

1. Closed chamber, five-digit balance (Mettler Toledo, model: AX105DR)

*Note: A five-digit balance allows for more precise measurements even if the root tissue mass is not ideal. Moreover, since water loss is gradual in small amounts of mass, a 4-digit balance might not capture these small changes within the timeframe of the assessment.*

2. Pre-calibrated humidity and temperature indicator (Digi-sense, Fischer Scientific UK LTD, catalog number: 15890036)

3. Digital Timer (FisherBrand^TM^, catalog number: 17951662)

4. Digital caliper 150 mm (RS PRO, UK, catalog number: 243-6808)

5. Drying oven (Mini 6L CLAD)

## Software and datasets

1. Microsoft Excel, included in the Microsoft 365 Apps; any open-source spreadsheet editor will work

## Procedure

**A. Digital balance setup**

1. Completely fill tea bags with self-indicating silica gel and tape them to the digital balance chamber. Place a temperature and humidity digital logger. Ensure that they do not disrupt the opening and closing of the closed chamber or the balance itself. Usually, 3–4 full tea bags are enough to maintain a constant relative humidity (Figure 1).

**Figure 1.**
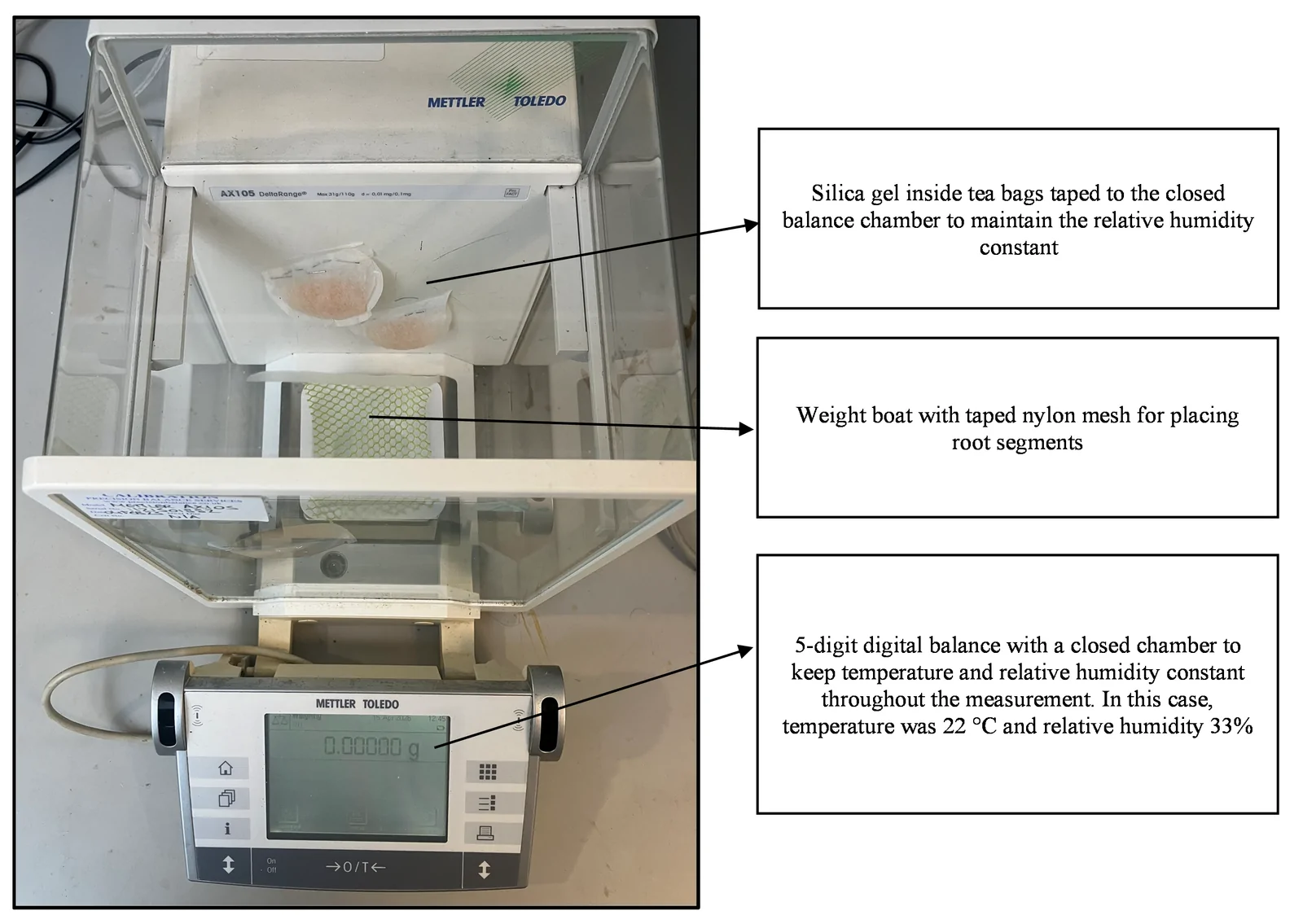
The digital balance setup shows the zeroing of the weight boat with the mesh where the root segments will be placed. Large fluctuations of temperature and relative humidity will impact on the rates of water loss; thus, it is important to keep the balance chamber in a temperature-controlled environment. Silica gel is taped inside the chamber and left for 10–20 min for the chamber to acclimate. The chamber should be opened and closed as quickly as possible when placing the samples to reduce the impact on the relative humidity at room temperature.

2. Tape a thin nylon mesh over the top of a weighing boat to support the roots whilst weighing. Ensure that the tape is at the edges of the mesh so that the root segments placed over the mesh are not in contact with the tape (Figure 1).

3. Place the weighing boat inside the balance chamber and zero the balance. Ensure that the balance chamber is closed and airflow is minimized (Figure 1).

4. Allow 10–20 min (or as needed) for the chamber to reach a stable relative humidity (30%–35%) and temperature. The balance should be at constant room temperature (20–25 °C).

**Critical:** Ensure that the balance setup is done before the root segments are ready to measure.

**B. Root preparation**

1. Take the rice roots out of their growth medium (e.g., loamy sand) and carefully clean with a fine paintbrush with deionized water (Figure 2a, b).

2. At the root region of interest, according to your experimental question, cut the roots of each seedling into root segments of the same length (Figure 2c). If possible, aim for at least 100–200 mg of fresh mass, and avoid segments with lateral roots. Longer root segments will increase the mass per segment and potentially reduce the number of segments needed. We suggest growing the plant you are working with and measuring a few root segments of different lengths before running an experiment. This way, you can predetermine the number of segments you will need for the actual experiment.

3. Measure the diameter of each root at the middle of the segment with a caliper, avoiding any damage to the root segment (Figure 2c).

4. Seal the cut ends of the segment with high vacuum grease so that you can see a visible seal, preventing water from being lost from the cut ends. A round dot of vacuum grease at the cut ends should be enough (Figure 2d). In our experience, if the root segment is not properly dry on the sides, the vacuum grease detaches easily. Test if this occurs before placing the segments in the balance and blot again on the cut ends of the segments before re-applying vacuum grease.

**Figure 2.**
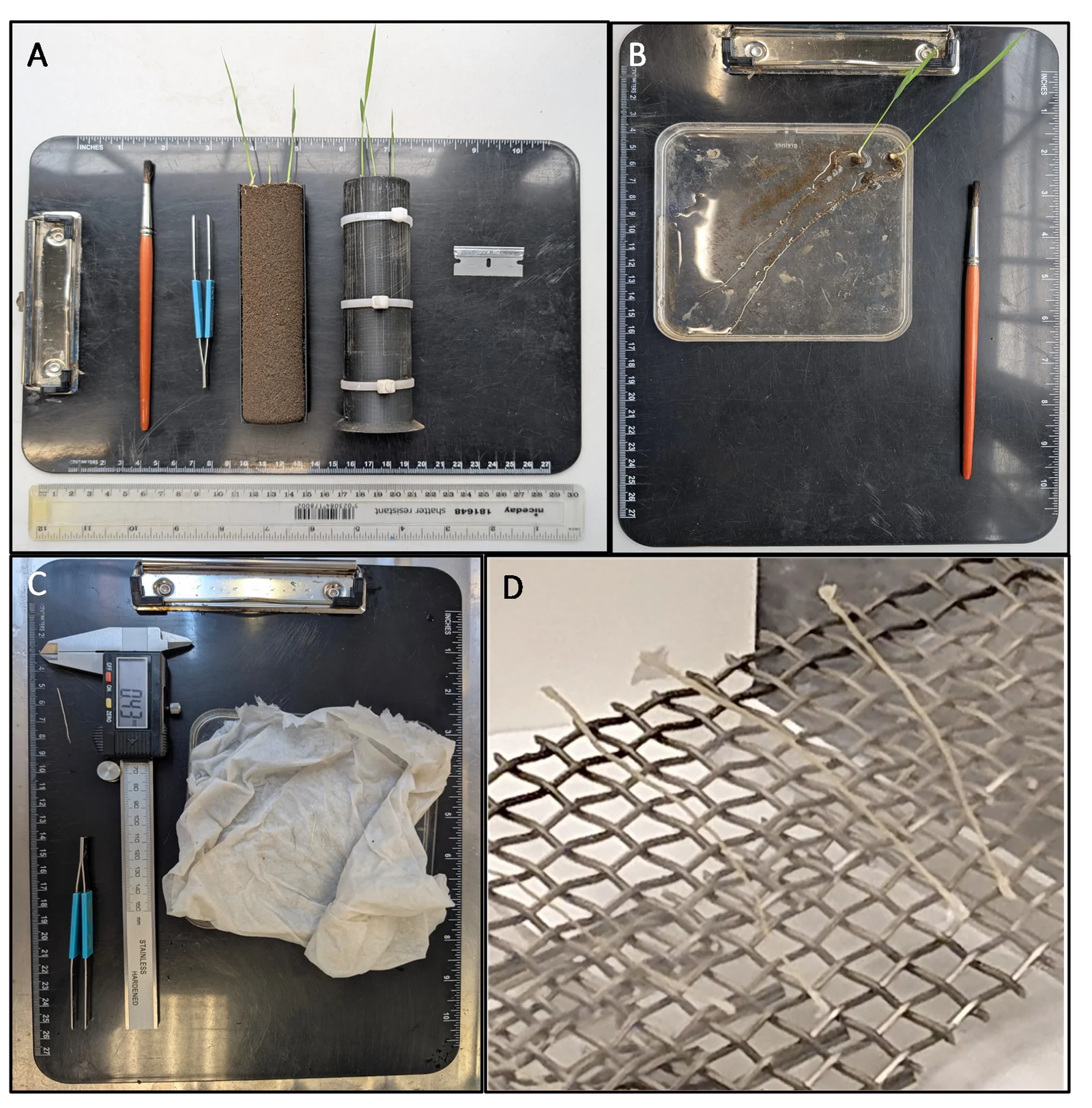
Tissue preparation for radial water loss measurements. (A) Extraction of roots from the soil column and (B) washing of the roots. Roots are gently removed from the soil by opening the soil columns and rinsing them with water. The loose plant material is then removed from the soil and transferred for further cleaning of soil particles using a soft brush around the area where the segments will be cut (e.g., 4–8 cm behind the root tip). (C) Preparation of root segments. The root region of interest is cut, and then 3 cm segments are prepared and kept in a moist paper towel. A digital caliper is used to assess the diameter of each segment. (D) Example of sealed root segments with vacuum grease inside a closed balance chamber. Notice the grease lumps on both ends of the segments. Another mesh was used for the example shown in (D).

5. Keep the root segments between moist paper towels at all times before moving them to the balance, avoiding submergence in water or drying out.

*Notes:*

*1. If the root segments have laterals emerging, cut the laterals as close as possible to the main root and apply a thin layer of vacuum grease over the cut to prevent additional water loss.*

*2. Roots of other species may be washed as presented here if they are of similar thickness. However, we recommend testing the cleaning process in your particular roots before moving to conduct radial water loss measurement, as different treatments might weaken the roots. Thinner roots, regardless of species or treatment, should be cleaned with added care.*

**Critical:**

1. Assessing root diameter with a caliper could squash/break the segment if the user is not careful, affecting the water loss assay. We thus suggest alternative means to determine root diameter:

a. Adjust the opening of the jaws of the caliper without the root segment between them, but rather on the side. Once you are satisfied with the opening of the jaws, attempt to place the segment between them. If it is too open, remove the segment and readjust the opening before inserting the segment again. If the segment does not fit between the jaws, open them again and repeat until the segment fits without forcing it.

b. Measure the root diameter close to the cut sites of the segment where vacuum grease is applied. Check if both ends are similar in diameter before assuming the whole segment’s diameter.

c. After cutting the root segment, obtain cross sections close to the cut sites of the root that are not part of the segment and determine their diameter using a microscope.

2. Ensure that all root segments are the same length; otherwise, the radial water loss calculations will not be accurate.

3. If the balance setup is not ready and you have already prepared the segments, keep the root segments constantly covered with moist paper towels, as water loss starts quickly once the segments are exposed to air.

**C. Weighing**

1. Place the prepared root segments onto the weighing boat, making sure that they do not touch, and that the high vacuum grease remains intact on the cut segment ends. If they fit in the weighting boat without touching each other, you can place as many segments as needed. However, the more segments you place, the more time is required for placing and repositioning the roots before you start noting down the mass, which is detrimental to the assessment.

2. Wait for the balance to equalize for 1–2 min inside the chamber before recording the initial fresh mass (g), starting the timer as soon as this initial fresh mass is recorded.

3. Record the weight every 30–60 s without disturbing the balance for at least 30–60 min or until 50% water loss is surpassed.

*Notes:*

*1. The silica gel must be replaced after every third replicate. This is important as it maintains the relative humidity inside the chamber at a constant 30%–35% at room temperature.*

*2. We consider all root segments pooled for one measurement as one replicate. We recommend at least four replicates per treatment.*

**D. Drying**

1. Following the measurements, create a small aluminum foil envelope to hold the roots securely, whilst still allowing space for water to evaporate (Figure 3). Record the foil envelope mass.

**Figure 3.**
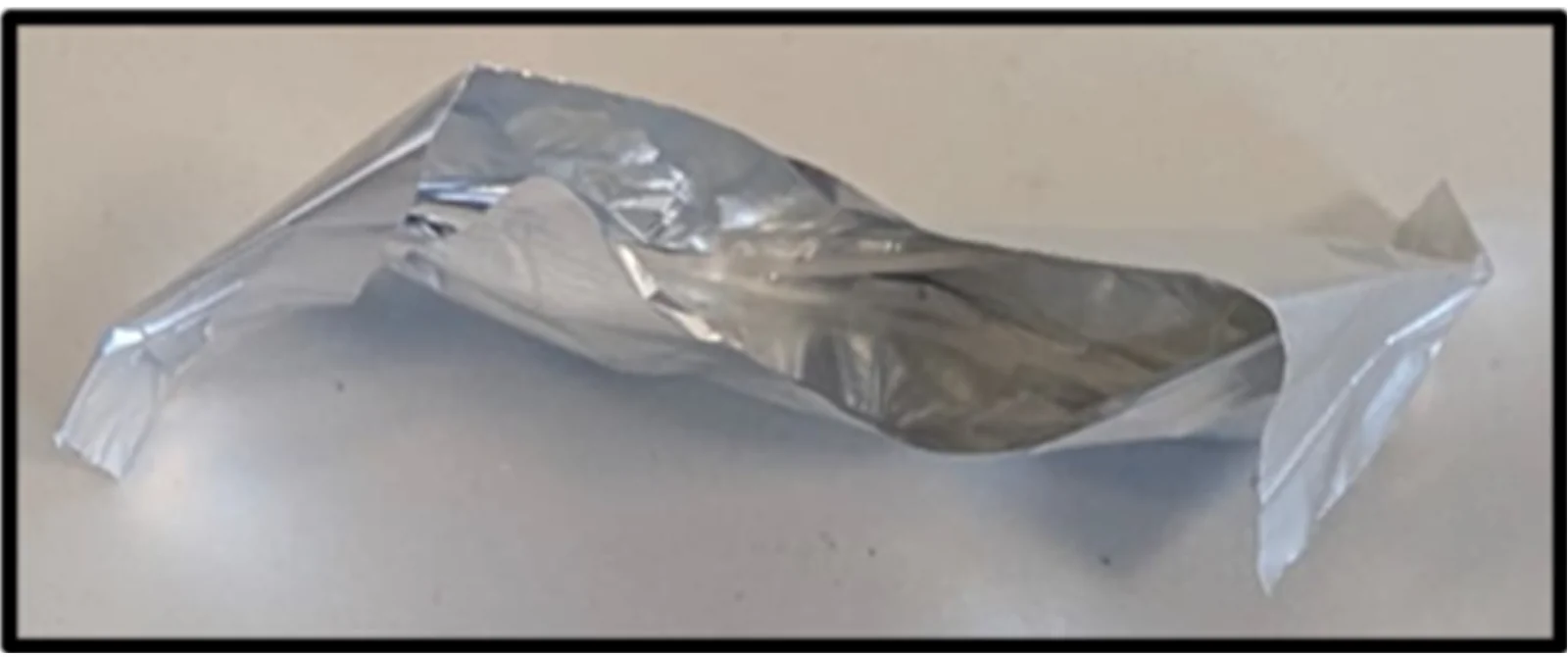
Example of an aluminum foil envelope. Ensure that the envelope can securely prevent the segments from falling while leaving space for water to evaporate for 48 h at 65 °C. Foil dimensions are 7 × 3 cm.

2. Place the root segments into the foil envelope and record the mass of the foil envelope with root segments.

3. Place the foil envelopes containing the root segments inside an oven set at 65 °C for 48 h to obtain the dry mass.

4. Remove the envelope from the oven, close it, let it acclimate to room temperature (a few minutes should be enough), and record the dry mass.

*Note: Ensure that the roots have dried completely. While 48 h should be enough, confirm this by measuring the dry mass at 48 h and again at 72 h. If the mass remains the same, the segments are completely dry.*

**E. Obtaining cumulated water loss and radial water loss values**

1. Calculate the water loss (g) between each time point [WL, e.g., the fresh mass (g) at time 1 minus time 0, then the fresh mass at time 2 minus time 1, etc.].

2. Subtract the foil envelope mass from the combined mass of foil envelope plus dried roots to obtain the root dry mass.

3. Subtract the root dry mass from the initial fresh weight (measurement of water loss at the start of the measurement) to obtain the total water content (TWC).

4. Calculate the cumulated water loss (CW) for each time point:

CWt_1_ (%) = WLt_1_ (g)/TWC (g) × 100 + CWt_0_

CWt_2_ (%) = WLt_2_ (g)/TWC (g) × 100 + CWt_1_

Repeat for all time points measured.

5. Convert the root length (*l*) of segments from centimeters to meters.

6. Average the root diameter (mm) of the segments used for a water loss measurement and convert to meters, e.g., if five segments were used for a replicate, average the diameter of those five segments.

7. Calculate the root radius (*r*) from the average root diameter.

8. Calculate the total lateral surface (TLS):

TLS (m^2) ^= 2π × *r* (m) × *l* (m) × number of segments averaged

9. Convert WLt_1...n_ to μmol and radial water loss (RWL):

μmol H_2_O t_1_ = WLt_1_ (g)/18.02 g/mol × 1,000,000

RWLt_1_ (μmol H_2_O m^2^/s) = μmol water t_1_/TLS (m^2^)/second per time point (s)

Repeat for all time points measured.

*Notes:*

*1. For the purpose of this method, we assume that the root segments are perfect cylinders. However, root segments are not completely cylindrical, nor do they present the same diameter along the whole segment (more so in longer segments).*

*2. The spreadsheet in Supplementary information (Table S1) includes calculations and additional notes to facilitate the calculation process.*

## Data analysis

1. Once you have obtained the radial water loss values for all the replicates (we recommend at least four), extract the RWL of each replicate for the desired time point when the target CW (e.g., 50%) was reached, and run statistical analysis (e.g., t-test, one- or two-way ANOVA).

*Note: There are no particular instructions for data analysis. We recommend you use your software of choice to run statistical analysis and perform typical tests to check for significant differences. In our experience, we utilize t-test and one- or two-way ANOVAs, provided the data fulfill the assumptions of such tests, and other requirements are met.*

## Validation of protocol

This protocol (or parts of it) has been used and validated in the following research article(s):

• Zhu et al. [8]. Single-cell transcriptomics reveal how root tissues adapt to soil stress. 473 *Nature* 642, 721–729 (2025). https://doi.org/10.1038/s41586-025-08941-z (Figure 4i–j).

## General notes and troubleshooting

**General notes**

1. The length of time that radial water loss is assessed will differ depending on the experimental question and species used. It is recommended that measurements continue until the root segments have lost at least 50% of their initial fresh weight, or for 30–60 min.

2. It is important to note that all segments used in one replicate should be cut to the same length.

3. If the root segment has a few large lateral roots, these can be removed using a razor blade, but must be sealed with high vacuum grease to prevent extra radial water loss from the lateral root cut site.

4. If the roots are thick enough, it is preferable to handle them with your fingers gently to avoid piercing them accidentally with tweezers (or use blunt tweezers). Take care at all times when doing this, to not squash or disfigure the root segments, particularly when measuring the root diameter with calipers.

**Troubleshooting**

**Problem 1:** Radial water loss fluctuates too fast or too slow.

Possible cause 1: Depending on the root tissue used, samples might lose water radially at varying rates, i.e., faster or slower. If the weight varies fast and gives highly fluctuating values, increase the sampling time, e.g., from 30 to 60 s. Contrarily, reduce the sampling time (e.g., from 60 to 30 s) if the changes in weight are too slow.

Solution 1: Run a test first to see how the roots naturally lose water radially over time, and then adapt the time at which you take measurements accordingly.

Possible cause 2: If the values are constantly drifting erratically, it could be due to vibrations/airflow surrounding the balance.

Solution 2: Ensure that the table or close surroundings where the balance is placed have no equipment causing vibrations, or other lab members working around the balance. Also, check that the balance chamber is properly closed and confirm whether air conditioning units are not pointing directly at it.

**Problem 2:** Roots shriveling up.

Possible cause: Leaving the roots to dry out by failing to keep them on moistened filter paper during preparation of the root segments.

Solution: All root segments should be in contact with a moist paper towel at all times and between each step during the preparation of root segments. Paper towels should remain moist and should not be allowed to dry out.

**Problem 3:** Vacuum grease not sticking properly to the end of the cut root segments.

Possible cause: The cut ends of the root segment are not blotted properly before applying vacuum grease, or the vacuum grease is not applied thoroughly.

Solution: Dry the cut ends of the root segment before applying vacuum grease. Place the vacuum grease on a spatula and then use this to manipulate the vacuum grease over the top of the cut end. There should be visible vacuum grease covering the end of any cut segment.

## Supplementary information

The following supplementary information can be downloaded here:

1. Table S1. Calculation spreadsheet with working example
